# Can Achievement Goals be Primed in Competitive Tasks?

**DOI:** 10.2478/hukin-2014-0026

**Published:** 2014-04-09

**Authors:** Iain Greenlees, Sean Figgins, Philip Kearney

**Affiliations:** 1 Department of Sport and Exercise Sciences, University of Chichester, Chichester, West Sussex, UK.

**Keywords:** priming, penalty-kicks, achievement goals

## Abstract

This study examined whether achievement goal priming effects would be observed within an overtly competitive setting. Male soccer players (N = 66) volunteered to participate in a soccer penalty-kick taking competition during which they took 20 penalty-kicks on 2 occasions. Following a pretest, participants were allocated to 1 of 5 priming conditions. Immediately prior to the posttest, participants in the priming conditions were asked to complete what was presented as an ostensibly unrelated task that took the form of either a computer task (subliminal priming) or wordsearch task (supraliminal priming). Results revealed that priming had no significant influence on performance.

## Introduction

Over the course of the last decade, researchers have demonstrated that, within sport, physical activity, and exercise contexts, an individual’s goal-directed behavior, affect, and cognitions can be influenced by priming (Ashford and Jackson, 2010; Banting et al., 2011; Bry et al., 2009; Friedman and Elliot, 2008; Radel et al., 2009; Sambolec et al., 2007). That is, goals such as cooperation, achievement, competitiveness can be activated (primed) in an individual by presenting environmental stimuli and/or words that are associated with, and activate mental representations of, that goal. This can be done either by presenting stimuli that individuals are not consciously aware of (subliminal priming) or that they are aware of but are unaware of the effects of the stimuli (supraliminal priming). For instance, in a recent study, Bry et al. (2009) showed that the sprint relay changeover times of novice performers could be enhanced by activating a co-operation goal using a scrambled-sentence task (a form of supraliminal priming that consists of asking participants to form a four word sentence out of five words that contain one or more words relevant to the goal to be primed).

Although this research offers the possibility that performance gains in sport may be achieved through the use of priming interventions (Sambolec et al., 2007) such a conclusion has recently been questioned by Winter and Collins (2013). In a study examining the efficacy of priming autonomous performance in field-hockey, they found no evidence for priming effects relative to a control condition. Winter and Collins argued against the use of priming interventions on a number of grounds including the applicability of primes in tasks requiring the performance of multiple subtasks, the difficulty of ensuring performers are unaware of priming interventions and the practical and ethical constraints of using priming techniques prior to performance. In addition, Winter and Collins questioned the extent to which the previous research in sport and exercise settings has provided a sufficiently ecologically valid examination of priming effects, pointing to the fact that most studies (barring their own and that of Ashford and Jackson (2010)) had not used expert performers.

An additional concern that exists with the ecological validity of current research is that no studies have examined the effects of priming in competitive sporting performance. Conceptually, it is possible to question the potential impact of priming goals such as competitiveness and achievement in competitive situations. Kay et al. (2004) argued that priming effects will only be seen when situational norms are somewhat ambiguous (e.g., when it is unclear whether one should co-operate or compete, as in the case of a negotiation or in a business meeting) but will not be seen when situational norms are unambiguous. Kay et al. (2004) provided support for this contention by showing that subjects who participated in the prisoner’s dilemma task when it was labeled, ambiguously, as “the situation” were influenced by a material prime reflecting competitiveness (pictures of objects drawn from the business world), whilst individuals who participated in the task when it was explicitly labeled as a community game were not. Thus, it would appear that when the situational demands are clear, as we may assume is the case in competitive events, priming goals in line with the situational demands may not exert the influence that is seen in many laboratory tasks in which the demands of the situation are ambiguous.

In opposition to this suggestion, research has also shown that priming can augment behavior in the presence of clear situational demands and existing goals. Aarts et al. (2005) proposed that priming not only activates behavioral goals (directs activity) but will also strengthen existing goals. They demonstrated that participants who had a preexisting goal to help others were more influenced by priming than participants who did not have such a goal. Additionally, Bargh et al. (2001) showed that priming a goal for cooperation did augment a consciously held goal to increase the amount of cooperative behavior engaged in. On the basis of this, it can be suggested that (a) goal priming may serve to augment existing goals that are made salient by the situation and/or are held by the individual, and (b) it may be possible, by extension, to enhance performance in competitive sporting events by strengthening goal-states such as high performance, achievement and competitiveness.

Conceptually then, it is unclear as to whether goal priming serves to augment goal states in competition settings given that competition is a salient, and unambiguous, feature of sport. Given this, the principal aim of the current research was to examine whether achievement priming enhances performance of skilled athletes in a competitive environment. The second aim of the research was to compare the effects of two forms of priming paradigm (subliminal versus supraliminal) used within the psychology literature. If priming interventions are to be developed in sport it will be important to understand which priming procedures are more effective. Although studies conducted by Srull and Wyer (1979) and Bargh and Pietromonaco (1982) have shown that supraliminal and subliminal priming exert equivalent effects, Bargh and Chartrand (2000) suggest that supraliminal priming tasks, where the individual is aware of the priming stimuli, yield stronger priming effects than subliminal priming tasks, as activation from a conscious, intentional processing of the primes is stronger than subconscious activation. Therefore, it was hypothesized that, if priming was observed to influence sporting performance, supraliminal priming would produce larger performance increases than subliminal priming.

## Material and Methods

### Participants

Sixty-six male, university level, soccer players (*M_age_* = 20.95 years, *SD* = 3.03; *M_years competitive experience_* = 12.49, *SD* = 4.32) from the University of Chichester, UK, responded to a campus wide advertisement to take part in a penalty-kick competition offering a £50 prize for the winning competitor (the participant who scored the highest score in any one of his two trials). The participants reported themselves to be of White British (93.8%), Black British-Caribbean (3.1%), Chinese (1.5%), and other (1.5%) ethnicity. All participants were volunteers and signed informed consent forms prior to participation. Institutional ethical approval was granted prior to the commencement of the study.

### Measures

*Performance.* Performance comprised of 20 penalty kicks towards an unguarded rectangular football goal with standard English Football Association dimensions (7.32 m × 2.44 m). All testing took place indoors. Shots were taken 10.97 m from the center of the target in accordance with standard football rules. The goal was divided into 13 zones and a rating system was employed based upon where the ball landed in accord with previous research using penalty-kick tasks (Ramsay et al., 2010). All penalties were taken in the presence of only the experimenter and a research assistant.

### Procedure

At the point of recruitment, participants were informed that the penalty-kick competition was part of a study examining the role of self-confidence in soccer and were given an individual testing time to attend the competition venue for the first session. On arrival at the first session, participants provided informed consent and demographic information and received an explanation of the experimental task. Afterwards, they completed a self-efficacy measure and then completed 10 practice kicks. Participants then took 20 penalty kicks. Following the completion of the first session participants were randomly assigned to either a subliminal achievement priming group (*n* = 14), a neutral subliminal priming group (*n* = 13), a supraliminal achievement priming group (*n* = 13), a neutral supraliminal priming group (*n* = 13), or a control group (*n* = 12).

One week after the first session participants returned to the testing venue. On arrival, subjects were informed by the experimenter that he was running behind schedule and there would be a five to ten minute delay. They were then approached by a confederate of the experimenter who explained that he was piloting some cognitive ability tasks for an unrelated research project and asked them to volunteer. Participants then went with the confederate to an adjoining room where they completed their assigned priming task. The subliminal priming task replicated the procedure used by Hart and Albarracín (2009). Participants completed a computer task where they were asked to determine as quickly as possible whether a string of letters (flashed on the computer screen) ended in a vowel or a consonant. Following fixating on the center of the screen for 2–7 s, participants were presented with a string of 13 letters (e.g., DYJNCBRDSTKQZ) for 150 ms, at which point a word relating to the priming condition (achievement: *attain, win, master, compete, excel, achieve, strive,* and *dominate,* or neutral: *stand, hat, stove, green, building, staple, lamp,* and *plant*) was flashed on to the screen for 33 ms. This was replaced by the original string of 13 letters for a further 20 ms followed by a string of six letters (e.g., POLKIM). Participants decided whether the string of six letters ended in a vowel or a consonant by pressing a designated key. A total of 75 presentations of the priming words for each condition were displayed in three sets of 25 primes.

The supraliminal priming condition took the form of a wordsearch puzzle task (Bargh et al., 2001). For both forms of the puzzle (achievement supraliminal *v.* neutral supraliminal) a 10 × 10 matrix of letters was presented, below which was a list of 13 words that were embedded within the matrix. Words could appear with letters in a straight line either from left to right or from right to left, reading down or reading up, and diagonally reading either down or up. Based on the recommendations of Bargh et al. (2001), each list contained the same set of six neutral words (*building, turtle, green, staple, lamp, and plant*), with the remaining seven words being manipulated based upon condition (achievement supraliminal or neutral supraliminal). In the achievement supraliminal priming condition these words were *win, compete, exceed, strive, attain, achieve,* and *master.* In the neutral supraliminal priming condition these words were *ranch, carpet, river, stove, robin, hat,* and *window.* The participants were required to find the words embedded within the wordsearch puzzle as quickly as possible. Participants in the control condition were informed that they were not needed for the validation test and asked to wait in the foyer of the testing venue until called to the room by the experimenter. The experimenter was unaware of the condition the participants had completed.

Once the priming task had been completed, participants returned to the competition venue and completed a self-efficacy questionnaire. They were then asked to warm-up and complete 20 penalty kicks. Following this, participants were individually debriefed. As part of the debriefing, the researcher probed participants for suspicions regarding the relationship between the two tasks with a funneled questionnaire protocol (Bargh and Chartrand, 2000). One participant expressed some suspicion about the experimental manipulation and was subsequently removed from the analyses. After the experiment, all participants were fully debriefed as to the nature of the study.

## Results

### Impact of priming on penalty-kick performance

Performance scores (Table 1) were analyzed by a 2 × 5 (trial × priming condition) ANOVA using the statistical package SPSS 19.0. Initial data screening indicated that assumptions of normality and homogeneity of variance were not violated. The ANOVA failed to show a significant trial by condition interaction effect, *F*(4, 60) = .16, *p* = .96, *η_p_^2^* = .014 or a main effect for group, *F*(4, 60) = .64, *p* = .64, *η_p_^2^* = .04. Therefore, priming was seen to have no influence on penalty-kick performance. A significant main effect for time did emerge, *F*(1, 60) = 5.60, *p* = .021, *η_p_^2^* = .09, indicating that penalty-kick performance improved from the pretest to the posttest.

## Discussion

The aim of the present study was to examine whether research findings that have shown that goal-priming enhances performance and cognitions in sport and exercise settings can be replicated within a competitive sporting environment. Although research has shown that priming can augment consciously held goals (Bargh et al., 2001), research has also shown that priming effects will disappear when the normative demands of a situation are unambiguous (Kay et al., 2004) and researchers have also questioned the utility of priming procedures in sport settings (Winter and Collins, 2013). We found that priming did not exert an influence on either performance scores in a penalty-kick competition, supporting Winter and Collins’ finding of no effect of priming, and these results thus call into question the idea that coaches and sport psychologists may be able to influence performance through the use of priming based interventions.

This finding demonstrates that priming effects should not be expected in competitive sport settings. Although, it has been proposed that existing goals will be strengthened through the use of primes (Aarts et al., 2005) and previous research has shown that priming can augment consciously held goals (Aarts et al., 2005; Bargh et al., 2001), these findings have only been observed in laboratory experiments in which conscious goals are assessed after the experiment has concluded or are activated via task instructions rather than in situations where the personal meaning and importance attached to the event results in the strong activation of goals (as may be the case in competitive sport). Additionally, sporting competitions may represent good examples of the types of situations with unambiguous normative demands which are not conducive to showing priming effects (Kay et al., 2004) and may be populated with individuals (with levels of chronic motivation to perform) who are resistant to the effects of priming (Hart and Albarracín, 2009).

Despite this conclusion, further research is warranted to more fully explore the potential uses of priming in overtly competitive situations. In addition to the limited sample size of the present study, a limitation with the current study is that we only sought to prime one type of goal, an achievement (or high performance) goal. Before the facilitative effects of priming in sport can be dismissed researchers need to examine different goal types. It can be proposed that priming procedures do have a positive impact on performance in competitive settings when goals other than those that may be chronically possessed by competitors or activated by situational demands (i.e., high performance or achievement) are primed. If situational characteristics (e.g., match importance, personal relevance) are high and so mean that priming achievement is redundant because such a goal is already held strongly, it may be possible, and indeed more appropriate, to use priming to activate goals that support the process of high performance. For instance, Ashford and Jackson’s (2010) research showed that hockey performance could be enhanced by priming autonomous performance (examples of priming words used were smooth, spontaneously, balanced and immersed) and Bry et al. (2009) showed that relay performance could be enhanced by priming cooperation (using phrases including *finding strength in numbers*). Thus, researchers should seek to explore the value of priming more subtle, process related goals in competitive environments.

The study also aimed to compare two forms of priming (subliminal *v.* supraliminal) most frequently applied within the psychology literature. Bargh and Chartrand (2000) suggested that supraliminal priming will produce stronger effects than subliminal priming. However, as the current results provide no support for the role of priming achievement goals in competitive situations, research is still warranted to examine the relative strengths of different forms of priming procedures in areas that have seen priming effects (e.g., in facilitating appropriate focus of attention, cooperation and autonomous motivation). This is an important area of future research potentially as the use of current supraliminal and subliminal procedures in sport are beset by potential ethical and practical concerns (i.e., withholding information about the nature of an intervention and the practicalities of asking competitive athletes to complete wordsearch puzzles prior to competition) and only one study has sought to examine the role of material priming in a physical activity context (Friedman and Elliot, 2008).

In conclusion, the current study found no support for the efficacy of priming as a means of eliciting enhanced performance in a competitive penalty-kick task and suggests that priming may be limited in its applicability to sport. This may call into question the suggestion that priming principles may be useful in shaping a new range of interventions (e.g., the manipulation of environmental objects, posters and team-talks) to prime motivated behavior. However, as research into the effects of priming in competitive sports is still very much in its infancy, the scope for further research in this area remains considerable.

## Figures and Tables

**Table 1:** Penalty-kick performance across priming conditions (mean values with standard deviations in parentheses)

Condition	*n*	Performance	
		Session 1	Session 2
Control	12	80.92 (27.40)	84.50 (28.34)
Neutral Subliminal	13	77.31 (29.67)	83.85 (25.82)
Neutral Supraliminal	13	79.92 (22.50)	89.92 (25.10)
Achievement Supraliminal	13	70.23 (28.23)	79.38 (26.79)
Achievement Subliminal	14	68.57 (24.23)	79.79 (18.49)

## References

[b1-jhk-40-245] Aarts H, Chartrand TL, Custers R, Danner U, Dik G, Jefferis VE, Cheng CM (2005). Social stereotypes and automatic goal pursuit. Soc Cognition.

[b2-jhk-40-245] Ashford KJ, Jackson RC (2010). Priming as a means of preventing skill failure under pressure. J Sport Exercise Psy.

[b3-jhk-40-245] Banting LK, Dimmock JA, Grove JR (2011). The impact of automatically activated motivation on exercise-related outcomes. J Sport Exercise Psy.

[b4-jhk-40-245] Bargh JA, Chartrand TL, Reis H, Judd C (2000). Studying the Mind in the Middle: A Practical Guide to Priming and Automaticity Research. Handbook of Research Methods in Social Psychology.

[b5-jhk-40-245] Bargh JA, Gollwitzer PM, Lee-Chai A, Barndollar K, Trötschel R (2001). The automated will: nonconscious activation and pursuit of behavioral goals. J of Pers Soc Psychol.

[b6-jhk-40-245] Bargh JA, Pietromonaeo P (1982). Automatic information processing and social perception: The influence of trait information presented outside of conscious awareness on impression formation. J of Pers Soc Psychol.

[b7-jhk-40-245] Bry C, Meyer T, Oberlé D, Gherson T (2009). Effect of priming cooperation or individualism on a collective and interdependent task: changeover speed in the 4 × 100-meter relay race. J Sport Exercise Psy.

[b8-jhk-40-245] Friedman R, Elliot AJ (2008). Exploring the influence of sports drink exposure on physical endurance. Psychol Sport Exercise.

[b9-jhk-40-245] Hart W, Albarracín D (2009). The effects of chronic achievement motivation and achievement primes on the activation of achievement and fun goals. J of Pers Soc Psychol.

[b10-jhk-40-245] Kay AC, Wheeler SC, Bargh JA, Ross L (2004). Material priming: The influence of mundane physical objects on situational construal and competitive behavioral choice. Organ Behav Hum Dec.

[b11-jhk-40-245] Radel R, Sarrazin P, Pelletier L (2009). Evidence of subliminally primed motivational orientations: the effects of unconscious motivational processes on the performance of a new motor task. J Sport Exercise Psy.

[b12-jhk-40-245] Ramsey R, Cumming J, Edwards MG, Williams S, Brunning C (2010). Examining the emotion aspect of the PETTLEP-based imagery with penalty taking in soccer. J Sport Behav.

[b13-jhk-40-245] Sambolec EJ, Kerr NL, Messé LA (2007). The role of competitiveness at social tasks: Can indirect cues enhance performance. J of Appl Sport Psychol.

[b14-jhk-40-245] Srull TK, Wyer RS (1979). The role of category accessibility in the interpretation of information about persons: Some determinants and implications. J of Pers Soc Psychol.

[b15-jhk-40-245] Winter S, Collins D (2013). Does priming really put the gloss on performance?. J Sport Exercise Psy.

